# Antiviral options and therapeutics against influenza: history, latest developments and future prospects

**DOI:** 10.3389/fcimb.2023.1269344

**Published:** 2023-11-29

**Authors:** Clement Meseko, Melvin Sanicas, Kumari Asha, Lanre Sulaiman, Binod Kumar

**Affiliations:** ^1^ Regional Centre for Animal Influenza, National Veterinary Research Institute, Vom, Nigeria; ^2^ Medical and Clinical Development, Clover Biopharmaceuticals, Boston, MA, United States; ^3^ Department of Microbiology and Immunology, Chicago Medical School, Rosalind Franklin University of Medicine and Science, North Chicago, IL, United States; ^4^ Department of Antiviral Research, Institute of Advanced Virology, Thiruvananthapuram, Kerala, India

**Keywords:** influenza virus, influenza therapeutics, antiviral drugs, epidemics, pandemics, anti-infectives, *in vitro* trials, clinical applications

## Abstract

Drugs and chemotherapeutics have helped to manage devastating impacts of infectious diseases since the concept of ‘magic bullet’. The World Health Organization estimates about 650,000 deaths due to respiratory diseases linked to seasonal influenza each year. Pandemic influenza, on the other hand, is the most feared health disaster and probably would have greater and immediate impact on humanity than climate change. While countermeasures, biosecurity and vaccination remain the most effective preventive strategies against this highly infectious and communicable disease, antivirals are nonetheless essential to mitigate clinical manifestations following infection and to reduce devastating complications and mortality. Continuous emergence of the novel strains of rapidly evolving influenza viruses, some of which are intractable, require new approaches towards influenza chemotherapeutics including optimization of existing anti-infectives and search for novel therapies. Effective management of influenza infections depend on the safety and efficacy of selected anti-infective *in-vitro* studies and their clinical applications. The outcomes of therapies are also dependent on understanding diversity in patient groups, co-morbidities, co-infections and combination therapies. In this extensive review, we have discussed the challenges of influenza epidemics and pandemics and discoursed the options for anti-viral chemotherapies for effective management of influenza virus infections.

## 1 History of development of anti-viral and chemotherapeutics

Viral infections, at times, cannot be prevented but consequent morbidity and mortality can be ameliorated with the use of selective anti-infectives in the management of clinical signs, sequelae and secondary complications. Although no antimicrobial by design is virucidal as obtainable for bacterial and other microbial agents, however anti-viral drugs act to limit viral load through inhibitory activities on replication pathway and egress. All steps in the life cycle of the virus starting with host cell entry, transcription, translation, and integration to the point of release from the host cell are steps that can be explored as molecular targets for antiviral therapy (Wang-Shick, 2017). The effect of the actions of these antagonists is a reduction in the burden of virus on host, and disease progression. While antivirals may not actively prevent infection, most of them are able to inhibit virus multiplication that allows the host innate immunity to cope with while infection runs its course (Raymund, 2011; Iwasaki and Pillai, 2014).

An era of viral chemotherapy began in 1950, when thiosemicarbazones was found to have antiviral effect on vaccinia virus when inoculated in fertile eggs and laboratory mice. However, efforts on global eradication of the variola virus through implementation of smallpox vaccination slowed down active research in the use of antivirals and further exploration of the antiviral compounds for orthopox (Hamre et al., 1950; De Clercq, 2010). However, in years that followed, breakthrough in the use of animal models, laboratory propagation of viruses, molecular biology and identification of viral enzymes that can be selectively inhibited, provided impetus for further anti-infective research. The major stimulus for the development of antiviral drugs happened when HIV/AIDS emerged in 1983, which resulted in the use of anti-retrovirals that targeted several viral enzymes (Littler and Oberg, 2005).

Successful use of anti-infectives in the management of infection with Human immunodeficiency virus (HIV), for instance, was achieved by inhibiting replication of the virus to reduced levels in the host and limiting its detection below the levels of significance in plasma during the course of the antiretroviral (ARV) treatments (Garcia et al., 2004). Successful management of HIV infection with either single or combination therapy (highly active antiretroviral therapy) have showed the potential impact of antivirals on over 12 million lives (Douglas et al., 2016). Other viral infections that have been successfully managed with selection of anti-infectives include Hepatitis, Lassa fever, Dengue and few tumorigenic agents. Although no antivirals have been licensed by the US Food and Drug Administration (FDA) for treatment of Ebola, an important re-emerging disease, hyper immune serum or convalescent plasma have been successfully used in the absence of effective antivirals or monoclonal antibodies (Kaner and Schaack, 2016; van Griensven et al., 2016). In the course of the largest outbreaks of Ebola virus disease (EVD) in West Africa, some trial anti-infectives were deployed. For instance, the World Health Organization, approved brincidofovir developed by a North Carolina-based company (Chimerix Inc.) for use in consenting and confirmed Ebola patients after the anti-viral was found to be effective in the laboratory against Ebola virus in experimentally infected cells. Unfortunately, all the four patients on trial treatment died of illness consistent with EVD (Dunning et al., 2016). Similarly, interferons, family of naturally occurring proteins already in use for treatment of hepatitis B and C, were also used in EVD treatment trials with variable outcomes (Sweiti et al., 2017). Interferons are substances produced by the host cell in response to viral infection and they have widespread potential as anti-viral including compounds that also serve as interferon inducers. Exogenous interferons have prophylactic anti-viral activity against infections such as vaccinia, rhinovirus, and influenza (Wendell, 1982). When interferon is used as anti-infective, it can inhibit infection by preventing viral entry into target cells. Interferons also act by blocking different stages of viral replication and are active against many viruses. In the West Africa Ebola outbreaks, Interferon beta particularly was described as potential reducer of the viral load, faster in patients compared to control group. Patients, who did not receive interferon, had significantly higher risk of dying compared to those who received the treatment (Konde et al., 2017).

**Figure 1:**
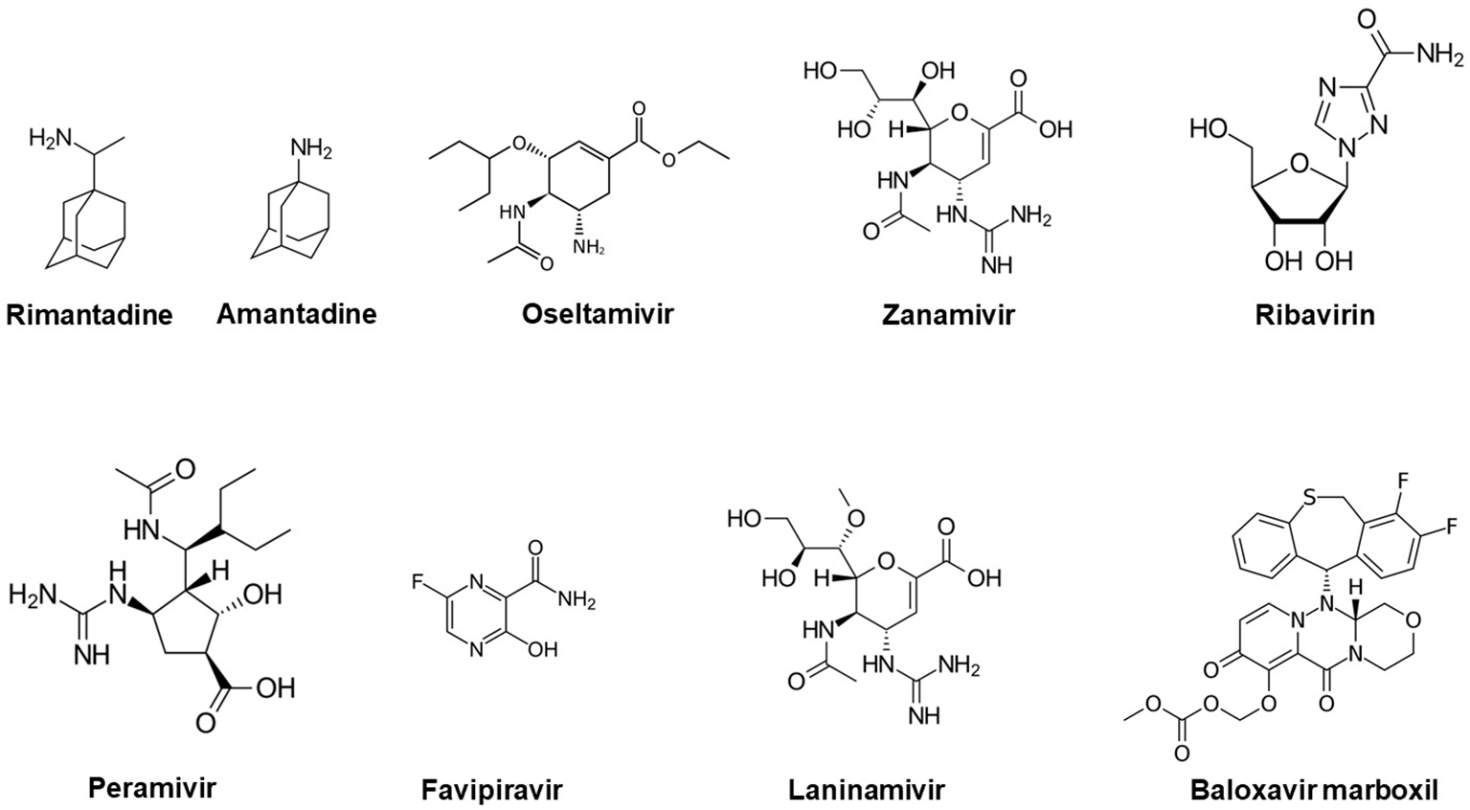
Chemical structures of common antiviral compounds used in the management of influenza infection.

Favipiravir (T-705; 6-fluoro-3-hydroxy-2-pyrazinecarboxamide) anti-infective, a pyrazinecarboxamide derivative (**Figure 1**) earlier developed as anti-influenza drug (Avigan) by Toyama Chemical, was successfully administered to a Cuban doctor who was infected by EVD in Sierra Leone. Besides influenza and other RNA viruses, the drug could inhibit a wide range of viruses including West Nile, foot-and-mouth disease (FMD), Nipah, Zika, yellow fever, flaviviruses, arenaviruses, enteroviruses, bunyaviruses, alphaviruses (Furuta et al., 2009; Furuta et al., 2013; Caroline et al., 2014). The oral drug acts through selective inhibition of RNA-dependent RNA polymerase during infection of many RNA viruses and was also approved for stockpiling to manage influenza pandemics in Japan in 2014. Other important anti-virals with proven efficacy against influenza virus include: Rimantadine, Amantadine, Oseltamivir, Zanamivir, Ribavirin, Peramivir, Laninamivir and Baloxavir marboxil (Naesens et al., 2016). Recent emergence of COVID-19, another important respiratory infection has also shown the importance of anti-viral options including naturally occurring anti-oxidants as complement to vaccine innovations (Forcados et al., 2021).

## 2 Emerging influenza threat and bio-risk reduction

Influenza infection, caused by influenza viruses, is common, seasonal, and global (Kumar et al., 2018; Asha and Kumar, 2019). The disease is transmitted by inhalation of aerosols containing virions via the respiratory mucosa (Iwasaki and Pillai, 2014; Asha et al., 2021). More than one hundred years ago in 1918, a pandemic emerged during World War I that killed more people than the arms battle. The catastrophe was caused by an influenza virus which was retrospectively identified as Influenza A/H1N1 (Oxford et al., 2002). The virus belongs to the family *Orthomyxoviridae* and causes respiratory disease that affects most animals and human of all ages but is reported to be more severe in the young, the aged and immune-compromised individuals. In a typical influenza season in the USA, illness and death are highest among adults aged ≥65 years, and children aged <2 years. Persons with pre-existing medical conditions are also at increased risk for influenza complications (Thompson et al., 2003; Thompson et al., 2004). The seasonality of influenza requires that susceptible persons including those with underlining infection, concurrent or co-infections are helped to manage complications. Annual vaccination is recommended for people with chronic underlying diseases and for 65 years or older individuals (Stiver, 2003). Many patient groups as well as occupationally exposed persons are also advised to get regular influenza vaccination and pre-immunity through annual vaccination prior to influenza seasons. This is in order to prevent development of clinical symptoms and spread of infection in the larger population.

Since 1918 and probably before that time, Influenza virus has continued to circulate in human with novel strains emerging and re-emerging from animal reservoirs. Unfortunately, the 1918 pandemic happened before the medical applications of chemotherapy and hence the usefulness of antivirals in managing clinical outcomes could not be appropriated. There was also lack of vaccine that could have prevented the pandemic, and hence about 50-100 million people died due to the impact of severe respiratory symptoms (Taubenberger and Morens, 2006). The virus subsequently caused more pandemics in the year 1957 (Asian flu) and in 1968-69 (Hong Kong flu) resulting in large number of deaths (Reid and Taubenberger, 2003). In 2009 another pandemic of influenza virus (A(H1N1)pdm09) emerged in Mexico. Although the pathogenicity of this novel strain was comparatively mild, nonetheless, around 151,700 –575,400 respiratory and cardiovascular deaths were linked to the infection that spread to 214 countries (Dawood et al., 2012). Before the 2009-H1N1 pandemic, there had been serious threats to public health from interspecies transmission of Highly Pathogenic Avian Influenza (HPAI)-H5N1 from poultry to human and 600 fatal cases out of 1000 infected people have been documented (WHO, 2018a). In 2013, subtype H7N9 emerged in China from poultry and caused waves of morbidity and mortality more in humans, than in birds where it exists in the form of Low Pathogenic Avian Influenza (LPAI). Till May 2018, over 1,564 laboratory-confirmed human infections with Avian Influenza A/H7N9 virus were reported (Gao et al., 2013; WHO, 2014). Patients who contracted influenza A/H5Nx and A/H7N9 infection were majorly treated with oseltamivir since the virus was resistant to the M2 blockers. The therapeutic effectiveness of neuraminidase inhibitors, however, can also be compromised due to the emergence of drug resistant variants (Marjuki et al., 2015).

In 2018, a patient was diagnosed with acute respiratory infection (ARD) requiring hospitalization and for the first time was associated with novel Influenza virus- A/H7N4 of avian origin. The 68-year-old woman was from Jiangsu province in China with pre-existing coronary heart disease and hypertension underscoring the higher susceptibly for immuno-compromised individuals (WHO, 2018b). Though it was alluded that the causative virus was sensitive to adamantanes and neuraminidase inhibitors, it is a reminder of active bio risk exposure to influenza from animals particularly in occupationally exposed groups (WHO, 2018b). It also highlights the important role that animal reservoirs play in seeding potential pandemics to human requiring bio-surveillance (Meseko et al., 2018). In many cases, human exposure arises from environmental and occupational handling and contact with animals, especially amongst farmers, veterinarians and traders at live bird markets (LBMs). Thus LBMs, poultry farms and other activities (games and recreations) that promote contact with animals remain hubs for virus amplification, persistence, dissemination and transmission to humans thus, requiring targeted anti-infective interventions (Mao et al., 2017). Similarly, the 2019 outbreak of SARS-CoV2 leading to the pandemic has revealed our preparedness and is an alarm that a lot more needs to be done for the management of future zoonotic outbreaks (Menéndez, 2022).

Influenza virus is yet the most potent bio-risk pathogen far above Ebola because of its ability for airborne transmission. The frequency of infections with seasonal influenza virus, continued outbreaks of the avian influenza A/H5Nx viruses in many countries, emerging novel influenza virus strains some with markers of antiviral resistance; underscore the importance of influenza virus research and prevention with potent vaccines. It is imperative to identify and stock pile effective anti-infectives to treat already infected population as a component of bio-risk management. This is desirable in saving life, preventing infection from spreading and for safety concerns in the larger society.

## 3 Management of Influenza through chemotherapeutics

Anti-infectives and antiviral therapy are promising treatment option to limit the duration and severity of influenza infection. They reduce negative impact of diseases often observed with endemic/seasonal influenza and occasional emergencies associated with pandemics. Since influenza viruses have several decades of history of causing pandemics, it is imperative to be prepared to manage its outbreaks before it spreads on a large scale and cross boundaries. Early treatment with antivirals can significantly bring down the risk for severe illness or death related to influenza, including from strains responsible for pandemics (Siston et al., 2010). Hence, treatment of influenza and associated symptoms within 48 hours of onset with traditional chemotherapies such as oseltamivir, a neuraminidase inhibitor (NAI), given at 75 mg daily, or twice in a day can be effective. Another effective anti-viral is amantadine (directed against viral M2 protein), which when given at 100 mg twice daily can be used both as treatment and prophylaxis (Salyer, 2007). In recent time however, relative antimicrobial resistance has been observed with some of the traditional and approved influenza antivirals (oseltamivir, zanamivir, amantadine, and rimantadine). Specifically, circulating A(H1N1)pdm09 strain that have now become seasonal is resistant to amantadine and rimantadine (CDC, 2010). Also in Japan in the course of 2013–2014 influenza season, cluster of influenza A(H1N1)pdm09 virus showed cross-resistance to the NAIs- oseltamivir and peramivir (Takashita et al., 2015). Some of the resistance by seasonal influenza viruses arise due to mutations in the viral polymerase gene through antigenic drifts and shifts. These generate hemagglutinin (HA) and neuraminidase (NA) antigenic variants giving rise to viral subtypes that can escape from pre-existing antibody-mediated immune responses (Iwasaki and Pillai, 2014).

Effective deployment of anti-infective would require continuous Influenza antiviral surveillance to detect emerging resistance. The susceptibility of neuraminidase inhibitor and influenza resistance information study (IRIS), are crucial for monitoring occurrences of resistance in influenza viruses. Antiviral resistance arising from genetic mutations and re-assortment (drifts and shifts), gives rise to novel subtypes or accumulation of mutations that presents changes in gene locus/protein domain and targets of chemotherapy. Sometimes interspecies transmission between human and reservoir animals accelerates emergence of a pool of variants that are resistant to currently used anti-infective to which parent virus were hitherto susceptible. It is therefore important not only to devise novel strategies or develop new generation of effective drugs but there is also an urgent need to understand the underlying mechanisms and forces that drive anti-infective resistance (Hughes and Andersson, 2015). Some antivirals against influenza are currently approved as mono therapy in uncomplicated infections. Clinicians may thereafter explore a number of options for combination therapy according to clinical presentation and response to treatment (WHO, 2018c). In intractable cases of influenza virus infections that may arise due to complications of co-infections, emergence of drug resistance strains, treatment options may include options may include increasing the dose of existing drugs (Davies, 2010; Dutkowski, 2010). In addition, combination therapies including the existing and/or novel antivirals, with distinct modes of action are very useful. In the foray also include ethno-pharmaceuticals derived from many plants and ethnic products that have been tested to reduce clinical symptoms and complications of influenza and other respiratory infections (Amber et al., 2017). The World Health Organization (WHO) listed about 21,000 medicinal plants, most of which act as immunomodulators with therapeutic potentials, as herbal, traditional/Indigenous medicines (Mahima et al., 2012). Some *in-vivo* studies have also shown the protective role of kolaviron (KV), a bioflavonoid isolated from Garcinia kola. As a natural antioxidant and anti-inflammatory agent, kolaviron has been shown to inhibit acetylcholinesterase activities in the hippocampus and stratum of wistar rats (Ijomone and Obi, 2013). In a challenge experiment on BALB/c mice (Awogbindin et al., 2015), it was suggested that KV may be effective in delaying clinical symptoms of influenza by mechanism unrelated to those used by existing anti-influenza drugs but closely associated to its antioxidant and immunomodulatory functions. Similarly, the extract of the root-bark of the African Baobab tree *(Adansonia digitata Lin)* was found to possess antiviral property on respiratory virus of poultry and may be useful in managing symptoms of influenza (Sulaiman et al., 2011).

Studies have shown that population susceptible to influenza infection include solid organ transplant and recipient of hematopoietic cell. These group are at higher risk of disease severity and developing complications, prolonged viral shedding, emergence of viral resistance, and deaths compared to the general population (Kmeid et al., 2016). Novel approaches to anti-infectives may be exploring gene therapy that may allow autologous transplantations of a patient’s own genetically corrected stem cells. This could be in the same vein with methods that efficiently add new copies of relevant gene to the hematopoietic stem cells leading to the safe and effective treatments for several primary immune deficiency diseases (Kohn and Kuo, 2017). Gene editing options may also attempt to identify alleles responsible for the production of metabolites, which are either deleted or modified for better chemotherapeutic response. Genome editing techniques is currently employed to construct viral mutants, prevent virus infections, eradicate proviral DNA, and to inhibit viral replication in infected cells (Okoli et al., 2018).

The changing map of viral chemotherapy underscores the need for new approaches in viral anti-infectives. A better understanding of the impact of mutations that result in antiviral resistance would enhance therapy of critically ill patients. In countering antiviral resistance to therapy, knowledge of potential permissive amino acid mutations in circulating viruses makes anticipation of emergence of non-compromising antiviral resistance feasible (van der Vries et al., 2013). Influenza viruses have the propensity for continuous antigenic shift and drift and the emergence of novel strains from animal reservoirs with decrease in susceptibility to antivirals. Influenza surveillance data on antiviral resistance patterns is therefore necessary to evaluate case by case susceptibility and selective clinical efficacy of antiviral. Anti-viral options and development of new antiviral drugs is a continuous science of discovery and selection of new anti-infective agents that are safe, effective, and affordable in the management of influenza epidemics and pandemics.

## 4 Viral chemotherapies: balancing efficacy and safety

Vaccination is one of the most practical and effective way to provide protection from influenza infections. The strategy however appears a little slow when there is a rapid spread of virus during pandemic situations. Although the vaccination is prophylactic in nature, they may not be very effective in the elderly, the immunocompromised and the infant high-risk groups, thus alternative strategies to develop safe and potent chemotherapeutic anti-influenza agents are needed in time.

**Figure 2:**
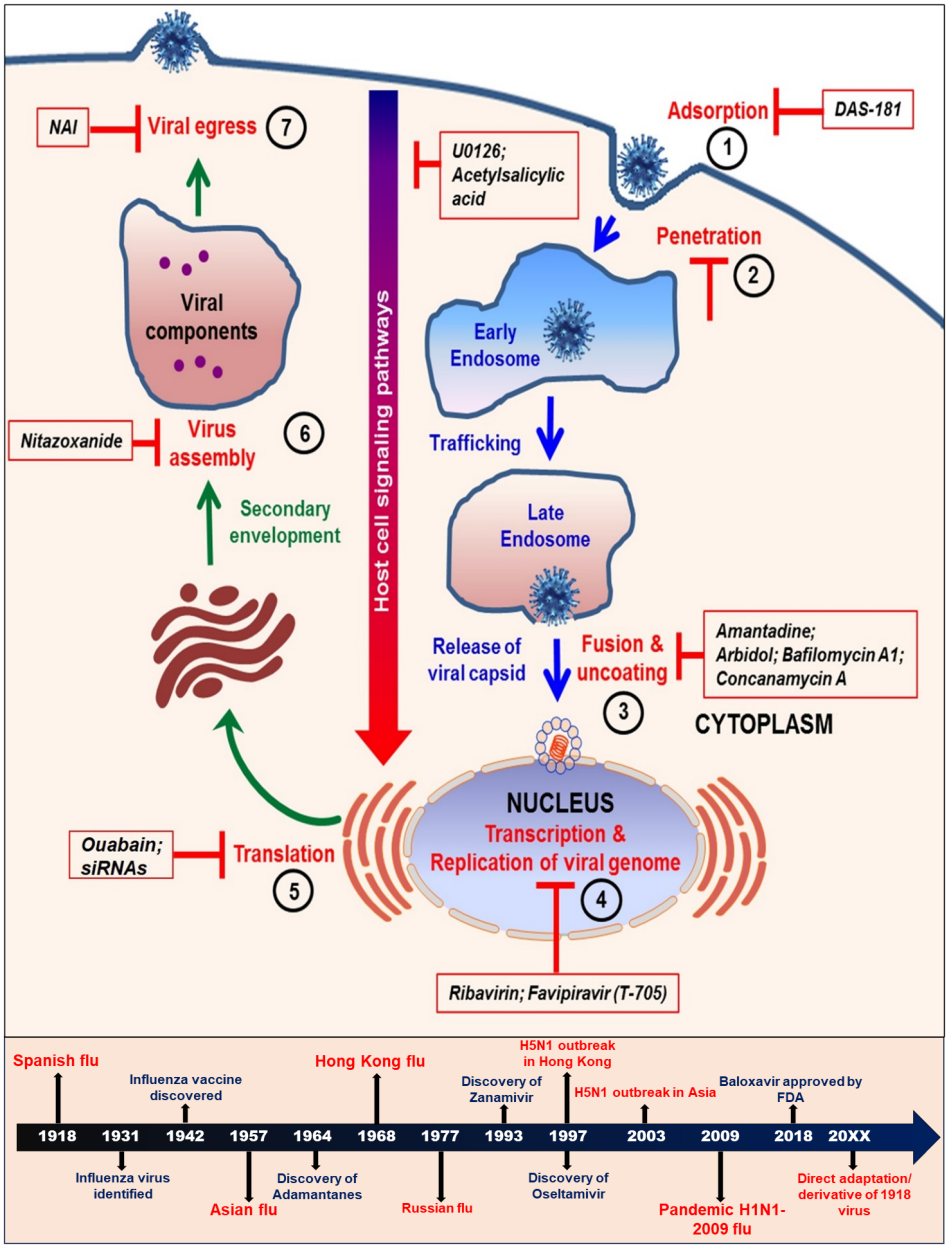
Schematic diagram showing various steps in the life cycle of influenza virus. The steps 1 to 7 are crucial targets for some of the currently used antiviral strategies. The lower panel shows the time lines of development of major antiviral drugs and occurrences of influenza pandemics.

An efficient chemotherapeutic drug should inhibit viral replication selectively without being detrimental to the host when used at a standardized concentration. Despite some current limitations, existing chemotherapy offers a better approach to the control of virus and has been formally licensed to be widely used against specific viral infections. Originally it was thought to be very difficult to interrupt the viral replication cycle without affecting the host cellular metabolism, however, it is now understood that there are several steps in the viral replicative cycle that differs from the normal host cellular processes and thus can be potential therapeutic targets for chemotherapeutic intervention (**Figure 2**).

Several antiviral compounds are formally licensed for clinical use against influenza viruses. The clinical indications, dosage, and mechanism of action of major ones are summarized in **Table 1**.

**Table 1:** Chemotherapeutic drugs, year of approval, spectrum and their mechanism of action.

Drug/Intake	Virus	Activity spectrum	Mechanism of action	Antiviral resistance
Amantadine (Oral)	Influenza A virus	Viral penetration inhibitors	Disruption of M2 ion channel function, virus uncoating and assembly	Widespread
Rimantadine (Oral)	Influenza A virus	Viral penetration inhibitors	Disruption of M2 ion channel function, virus uncoating and assembly	Widespread
Oseltamivir (Oral)	Influenza A and B virus	Viral release inhibitor	Inhibits enzymatic activity of neuraminidase	Uncommon
Zanamivir (Inhaled, nebulized, intravenous)	Influenza A and B virus	Viral release inhibitor	Inhibits enzymatic activity of neuraminidase	Rare
Peramivir (Intravenous)	Influenza A and B viruses	Viral release inhibitor	Inhibits enzymatic activity of neuraminidase	Uncommon
Favipiravir/ T-705 (Oral)	Influenza A, B, C and other RNA viruses	Viral replication inhibitor	Inhibits the activity of RNA dependent RNA polymerase of influenza viruses	Not reported
Laninamivir (Inhaled)	Influenza A and B viruses	Viral release inhibitor	Inhibits enzymatic activity of neuraminidase	Rare
DAS181 (Inhaled)	Influenza A and B viruses	Viral penetration inhibitors	Sialidase activity that destroys receptor for HA	Not reported

Antiviral drug against influenza was first discovered and available in 1960s when the amantadine (an amino derivative of adamantine) was approved for treatment and prophylaxis of influenza A viruses with an efficacy of around 90% (Reuman et al., 1989). In the 1990s, another drug, rimantadine (an analog), was approved and showed fewer side effects than amantadine. Extensive mechanistic studies on the action of adamantines led to the discovery of a new influenza viral protein (M2) and studies on the mutants resistant to rimantadine demonstrated that all mutant strains had a mutation in the second splicing readout of the gene 7, encoding protein M2 (Lamb et al., 1981). The M2 protein that also acts as an ion channel, is responsible for the internal acidification of the virus which is essential at the early stage of viral trafficking in endosomes and releasing its genetic material. The amantadine and rimantadine increases the pH inside the endosomes by inhibiting the transport of protons. This event hampers the separation of viral ribonucleoprotein (RNP) from M1 protein and subsequently on the onset of viral genome transcription (Hay, 1996). Both drugs were active against influenza A viruses and blocked their replication in both cell lines and animal models, however, viral resistance soon developed against them and they produced marked side effects (Arruda and Hayden, 1996; Shigeta, 1997). Studies further showed that the therapeutic efficacy of amantadine and rimantadine was observed to be insignificant in infected individuals as well as prophylaxis of healthy volunteers of a professional staff group (Belshe et al., 1989; Hayden et al., 1989). While it may take 1 to 2 weeks for the influenza strains to acquire resistance in animal models and cell lines, it may take just 2-4 days in humans after beginning of the amantadine and rimantadine chemotherapy. Other important problems while administering amantadine includes serious side effects such as dyspepsia, anorexia, hallucinations, and sleeplessness. Studies involving rats have revealed that amantadine also provided evidence of embryotoxicity and teratogenicity thus limiting its usage in cases of hepatic and renal diseases and in pregnant women (Horadam et al., 1981; Dolin et al., 1982; Reuman et al., 1989). The side effects due to rimantadine were similar but less noticeable than those of the amantadine (Dolin et al., 1982). The surveillance studies for amantadine and rimantadine resistant A (H3N2) viruses revealed that the global frequency of resistance increased from 0.8% during 1991-95 to 12.3% in 2004 and a after a year to 96%, 72%, and 14.5% in China, South Korea, and the United States, respectively (reviewed in (Kumar et al., 2018)) (Bright et al., 2006). In Russia, the analogs of amantadine (deitiforin) and rimantadine (adapromine), was recommended owing to their broad spectrum of antiviral activity as compared to rimantadine. The adapromine not only inhibited influenza A and B viruses but also the rimantadine-resistant strains. Although the efficacy was comparable to rimantadine, they both were also associated with side effects.

In late 1990s, the second generation of anti-influenza drugs called the neuraminidase inhibitors (NAI), were approved, and found to act against influenza A viruses. The 2 major NAIs used for treatments were the oseltamivir (widely used) and zanamivir (lower patient acceptance rate). Oseltamivir gained more popularity and became the choice of anti-influenza drug throughout the world. The drug inhibits the activity of viral neuraminidase thus preventing the viral budding and thereby restricting infection in respiratory tract (Treanor et al., 2000). The clinical investigation data further revealed that administration of oseltamivir decreased the disease duration by 30% (2.9 days), when the treatment was started in the first 36 hours after appearance of first symptoms, and the side effects such as sinusitis and bronchitis were significantly low (Treanor et al., 2000). A study based on large number of patients showed that oseltamivir treatment decreased the duration of disease to 3.1 days when administered within 12 hours of onset of symptoms as compared to 5.3 days when administered within 48 hours of onset of symptoms (Aoki et al., 2003). The studies based on resistant viruses revealed that while only 1 to 2 passages were sufficient to generate resistant strains due to amantadine and rimantadine pressure, it took several more passages for the virus to become resistant under the pressure of oseltamivir. The mutation was observed to be in the neuraminidase (NA) and in the viral HA that binds to the sialic acids (Mendel and Sidwell, 1998).

Zanamivir, another 2^nd^ generation NAI, was shown to inhibit the activity of neuraminidase of both influenza A and B viruses in cell line (von Itzstein et al., 1993). The *in vivo* models further validated its activity in mice and rats infected with influenza viruses (Hayden et al., 1997). It was also shown that the drug acted best when administered through the intranasal, intraperitoneal, or intravenous routes and the activity was absent upon oral administration since the drug could not be absorbed in the gastrointestinal tract and did not reach the respiratory tract (Hayden et al., 1997). Several other studies based on large number of samples proved the effectiveness of zanamivir in preventing the development of influenza infection significantly and also reducing the time of manifestation of symptoms (Group, 1998; Calfee et al., 1999; Monto et al., 1999). Zanamivir has also showed low toxicity and no serious side effects in clinical investigations (Cooper et al., 2003). A comparative study on the usage of oseltamivir and zanamivir in the treatment of pandemic influenza revealed that both drugs had similar efficacy in terms of relief of symptoms and adverse effects, however, the zanamivir provided much faster relief in temperature normalization in patients (Tuna et al., 2012).

Of the 5 currently used, major anti-influenza drugs, only 3 NAIs (oseltamivir, zanamivir and peramivir) are approved for use by the U.S. Food and Drug Administration (US-FDA) (FDA, 2018). The M2 ion channel blockers (amantadine and rimantadine) are not recommended by US-FDA owing to increasing resistance of circulating influenza strains toward the drugs. The M2 blockers are also found ineffective against the influenza B and C viruses (reviewed in (Kumar et al., 2018)) (CDC, 2018). With each passing year, the influenza viruses have also evolved to withstand the effect of antiviral drugs by gaining resistance. The number of oseltamivir resistant influenza has increased tremendously over the last few years. The global circulation of oseltamivir resistant influenza A(H3N2) and seasonal A(H1N1) viruses raised serious concerns about the potency of the drug (Hayden, 2006; Hayden and de Jong, 2011). The most recent example of oseltamivir resistance is the pandemic strain A(H1N1) pdm09 viruses (Hurt et al., 2012).

Another drug called the “Umifenovir (Arbidol)” gained popularity in Russia and was recommended by the Russian State Pharmacopoeial Committee for the effective treatment and prophylaxis of influenza A and B viruses in both adults and children. The broad-spectrum drug hindered viral replication and its immunostimulant and interferon-inducing activity and was better than the amantadine and rimantadine (Reviewed in (Leneva et al., 2004)) (Brooks et al., 2012; Leneva et al., 2016). Large population studies showed a high efficacy of the drug arbidol in reducing duration of the disease as well as reducing the time of symptoms such as headache, cough, rhinitis, and weakness in both adults and children (Kiselev et al., 2015). Recent studies also showed that arbidol inhibited both seasonal and pandemic H1N1 stain by modulating the expression levels of inflammatory cytokines to reduce viral replication and acute inflammation (Liu et al., 2013). The arbidol has also been shown to inhibit other virus infection, such as zika virus entry, as reported in recent studies (Fink et al., 2018). The world has, by now, seen a changing influenza antiviral landscape, and urgently need the development of novel chemotherapeutics against both emerging and remerging influenza viruses.

## 5 Potency of influenza antivirals

The global presence of oseltamivir resistant H3N2 and H1N1 strains are the signs of the limitation of antivirals against influenza viruses. There is therefore an urgent requirement for development of new antivirals with novel mechanisms of action and newer drug combinations to effectively manage the outbreaks of influenza. The development of novel NAIs and other pharmacological groups have shown promising outcome towards better management of influenza infections in adverse situations.

### 5.1 Neuraminidase inhibitors

Although oseltamivir is still recommended for treatment of influenza infections globally, there are more recently developed NAIs such as the peramivir and laninamivir which were approved for use in Japan in 2010. The US-FDA first issued an emergency use authorization (EUA) for the usage of peramivir in 2009-2010 pandemic period based on the safety data in clinical trials, and in December 2014 approved it for the treatment of influenza infections in adults (FDA, 2014). It was approved for use in both Japan and South Korea since 2010. It was approved for an intravenous administration in patients with acute uncomplicated influenza. Although it is high cost as compared to oseltamivir and the route of administration made it less popular in patients with mild infections, yet it is very useful for the critically ill patients or patients who are unable to tolerate other administration routes (Alame et al., 2016). Peramivir has been shown to bind to neuraminidase (NA) enzyme more tightly as compared to other NAIs and restricts the growth the both influenza A and B virus in *in-vitro* studies (reviewed in (Hata et al., 2014)).

Laninamivir is comparatively a new NAI with structural similarities with zanamivir. The drug has long retention time and a single dose is sufficient for one week unlike zanamivir or oseltamivir which needs to be administered twice daily (Ishizuka et al., 2010). The drug became highly popular in Japan due to its ease of single-dose administration. It could treat patients infected with seasonal influenza including those resistant against oseltamivir as revealed in a clinical trial (Watanabe et al., 2010). Compound 23b shows a potent inhibitory activity against neuraminidase from H5N1 subtype but nontoxic to MDCK cells (Wang et al., 2020).

### 5.2 Drugs acting against other viral components

Several small organic compounds such as quinones (Larson et al., 2012), an antibiotic stachyflin (Yoshimoto et al., 1999) and derivatives of benzamide and podocarpic acid (Luo et al., 1997; Staschke et al., 1998) have been shown to be effective in blocking the hemagglutinin mediated membrane fusion during influenza virus infection. They have been shown to have strain specificity and associated cytotoxicity in *in-vitro* models. A new drug called the DAS181, is a novel HA inhibitor that prevents virus attachment to epithelial cells by enzymatically removing the sialic acid receptors (Malakhov et al., 2006). The drug is effective even at nanomolar concentrations with minimal cytotoxic effects and is effective against both influenza A and B viruses (Moss et al., 2012). Since the drug removes both the human-like α2,6- and avian-like α2,3-linked sialic acids from cellular receptors, it acts as a broad range sialidase that works even for the viruses resistant to adamantines and NAIs. Nitazoxanide is a thiazolide compound that is known to have antiviral activity against influenza and other RNA viruses. They work mainly by inhibiting the influenza virus replication by hindering the maturation of hemagglutinin protein (Rossignol et al., 2009). Favipiravir (T-705) is another antiviral that has inhibitory activity against both NAI and adamantine resistant viruses by inhibiting the influenza RNA polymerase (Furuta et al., 2009). It is effective against all the influenza virus types (A, B, C) at very low concentrations and even works for other RNA viruses at higher concentrations (Furuta et al., 2009). Several other therapeutic options against influenza such as the CHCl_3_ extract of *Ferula assa*-*foetida*, pyrazole-based compound BPR1P0034, human monoclonal antibody termed A06, have been shown to be potent and beneficial as revealed in different studies (reviewed in (Khanna et al., 2012)). VX-787, a viral PB2 inhibitor, has been revealed to work against several strains of influenza A viruses and is effective both as prophylaxis and treatment (Byrn et al., 2015). Another potential antiviral drug candidate 1,3-dihydroxy-6-benzo [c] chromene (D715-2441) was shown to specifically bind to the PB2 protein thus significantly reducing the action of influenza RNA polymerase of the H1N1, H3N2, H5N1, and H7N9, as well as oseltamivir-resistant strains having H274Y NA mutation (Liu et al., 2018). This drug was also shown to provide a synergistic antiviral effect when used with zanamivir (Liu et al., 2018). Nucleozin and Naproxen are the nucleoprotein (NP) inhibitors and have shown antiviral activity mostly by inhibiting the NP binding to RNA in influenza A viruses (Cheng et al., 2012; Lejal et al., 2013). Certain influenza-NS1 inhibitors such as NSC125044 and Baicalin, have been shown to act as inhibitors of viral replication by re-establishing the antiviral effect of interferon and altering the binding domain of the NS1 protein respectively (Jablonski et al., 2012; Nayak et al., 2014). A recent study has demonstrated the promising activity of an antiviral compound FA-6005 that specifically targets the amino acid residue 41, and inhibits the activity of the vRNP complex of both influenza A and B viruses. The drug puts hindrance at various stages in the life cycle of influenza virus by reducing the NP/vRNP export, impairing the trafficking of circulating RNPs in the cytoplasm, hampering the virus uncoating process and disrupting the budding of daughter virions (Yang et al., 2021). OA-10, another newly discovered triterpene out of 11 oleanane-type derivatives, showed potent antiviral activity against replications of the IAV H1N1, H5N1, H9N2 and H3N2 in lung cell cultures (Ye et al., 2020). The A77 1726 also inhibited the replication of three IAV subtypes (H5N1, H1N1, H9N2) in multiple cell types (Wang et al., 2020).

Several studies have also shown the effect of short interfering RNA (siRNAs), ribozymes and DNAzymes that specifically targets the vital genes of influenza viruses to block their replication in cell line and mice models (Kumar et al., 2012; Rajput et al., 2012; Kumar et al., 2013; Asha et al., 2018).

### 5.3 Drugs acting against host cell factors to block viral infection

There are several drugs that are known to target the host cellular factors to block the influenza virus infection at several stages of infection. DAS181 is a sialidase that cleaves residual sialic acid from the cell surface of cells thus preventing the virus entry into target cells. A polypeptide, Aprotinin, prevents host proteases that cleave viral HA, thus interfering with viral binding and fusion as shown in an *in vitro* as well as *in vivo* study (Ovcharenko and Zhirnov, 1994; Zhirnov et al., 2011). Similarly, Bafilomycin A1 and Concanamycin A are antibiotics that dampen the process of endocytosis and fusion by inhibiting vacuolar H+ATPase activity that pumps protons from the cellular cytoplasm to the interior of the endosomes (Muller et al., 2011). Further, the inhibitors of key pathways such as the Raf/MEK/ERK pathway (U0126) and NF-κB transcription factor pathway (Acetylsalicylic acid) play crucial roles in reducing the viral load in experimental studies mainly by reducing the nuclear export of viral ribonucleoprotein (Mazur et al., 2007; Droebner et al., 2011).

### 5.4 Antiviral combinations and small molecular therapy against influenza

The constant evolution of influenza viruses and rapid resistance development against the existing antivirals has become a global concern and part of the broad subject of Antimicrobial Resistance (AMR). Thus, to overcome the limitations of monotherapy and to combat the emerging drug-resistant strains, a combination treatment seems a better therapeutic option. A combination therapy with one or two or more drugs targeting different viral proteins or host proteins may prove to be a superior and potent option to treat influenza infections (**Figure 2**) (Dunning et al., 2014). Small molecule combination therapy is an exciting alternative option for the treatment of influenza as it comes with an advantage of enhancing the overall efficacy and possibility of lower individualized dosages thus increasing the tolerance on patients. The combination therapy also supports reduced drug toxicity. It can either target the same viral protein, two or more viral proteins of influenza or it can also target host and virus associate molecular mechanisms (Shen et al., 2015; Batool et al., 2023).

A summary of several combinations of drugs with the subtype of influenza virus is shown in **Table 2**.

**Table 2:** Combinatorial approach towards management of influenza infections.

Drug combinations	Influenza subtype	Activity spectrum	Experimental condition	Ref.
Double drug combination				
Rimantadine + Ribavirin	H1N1 H3N2	Virus yield inhibition	*In vitro*	(Hayden et al., 1980)
	H1N1 H3N2 B	Virus yield inhibition	*In vitro*	(Hayden et al., 1984)
Amantadine + Ribavirin	H5N1	Virus yield inhibition	*In vitro*	(Smee et al., 2009)
	H1N1	Virus yield inhibition	*In vitro*	(Nguyen et al., 2010)
	H5N1	Virus yield inhibition	*In vivo*	(Smee et al., 2009)
Zanamivir + Rimantadine	H1N1 H3N2	Virus yield inhibition	*In vitro*	(Govorkova et al., 2004)
Oseltamivir carboxylate + Amantadine	H1N1 H3N2 H5N1	Virus yield inhibition	*In vitro*	(Ilyushina et al., 2006; Nguyen et al., 2010)
	H1N1 H3N2 H5N1	Virus yield inhibition	*In vivo*	(Masihi et al., 2007; Smee et al., 2009)
Oseltamivir carboxylate + Ribavirin	H1N1	Virus yield inhibition	*In vitro*	(Nguyen et al., 2010)
	H1N1 B H5N1	Virus yield inhibition	*In vivo*	(Smee et al., 2006; Smee et al., 2009)
Oseltamivir + Favipiravir	H1N1 H3N2 H5N1	Virus yield inhibition	*In vivo*	(Smee et al., 2010)
Tripple drug combination				
Oseltamivir carboxylate + Amantadine + Ribavirin	H1N1 H3N2 H5N1	Virus yield inhibition	*In vitro*	(Nguyen et al., 2009; Nguyen et al., 2010)

## 6 Reverse genetics, gene therapies, new approaches and prospects

Reverse genetics is a versatile technique which can be used to, among other things, generate re-assortant viruses. This can be done by transfection of cells with plasmids that contain viral genome, or through the combination of plasmids that code for different viral sequences and aided by helper virus (Stobart and Moore, 2014). New viruses can thus also be generated from cloned cDNA that contain point mutations, thereby capable of substituting gene segments, giving rise to a new virus strain different from the original parent virus (Neumann et al., 2002).

The influenza virus genome is highly segmented, and this makes it easy for strains to exchange RNA segments, thereby producing a virus different in antigenicity, pathogenicity, and other important biological characteristics. This also makes it easy for nucleotide substitution during gene replication (Webby and Webster, 2001), thereby leading to frequent antigenic drift and antigenic shift and this accounts for Influenza viruses’ abilities to produce epidemics and pandemics around the world. Thus, the possibility for the Highly pathogenic avian influenza H5N1 and the pandemic influenza have great possibilities to produce human infections, thereby necessitating a different approach to vaccine production and efficacy.

For the annual influenza vaccination, the vaccine is usually developed by the reassortment of the viral backbone segments of a highly productive influenza vaccine strain (donor) the hemagglutinin and neuraminidase segments from the virulent epidemic strain. This method involves a laborious screening of vaccine strain re-assortants but with the advent of reverse genetics, researchers are now able to design and produce influenza viruses of the required genotypes. Currently, there are several methods to do this, all based on reverse genetics (Jung et al., 2010; Engelhardt, 2013). These methods can be broadly divided into *helper-virus dependent* and *helper-virus independent* methods.

The helper-virus dependent methods include ribonucleoprotein transfection, nucleic-acid ribonucleoprotein reconstitution and viral vector-based ribonucleic acid reconstitution. The helper-virus independent methods, on the other hand, include *plasmid-only reverse genetics system*, *ribonucleoprotein transfection-based helper virus independent reverse genetics system* and *viral vector-based helper virus independent reverse genetics system.* Reverse genetics has therefore provided vaccine researchers with new techniques for designing vaccines against influenza viruses and proffering better preparedness against influenza pandemics. The versatility of this technique is shown by a team in Arizona which used a one-plasmid reverse genetics system to create a plasmid in which eight cassettes were ligated, giving profound advantages in generating recombinant influenza viruses for vaccine production as well as other important studies like virus characterization (Zhang et al., 2009). The success of these techniques and the attendant opportunities notwithstanding, influenza vaccine production system is still recommended to be cell culture or egg-based, with requisite quality controls (WHO, 2003).

### 6.1 Reverse genetics to understand pathogenicity factors

Reverse genetics can be used for a myriad of studies in vaccinology apart from the production of influenza vaccines. There is the need to understand the pathogenicity factors of influenza viruses and reverse genetics is making these studies much easier to accomplish. The roles of the influenza proteins like hemagglutinins, neuraminidase, polymerase basic proteins, polymerase acidic protein, nuclear export proteins and others, and the effect of any kind of mutations on the genes coding for these proteins have been well studied with the use of reverse genetics techniques (de Jong et al., 2006; Conenello et al., 2007).

### 6.2 Reverse genetics in understanding of influenza immunity

Understanding how influenza viruses attack and respond to host specific immunity to ensure survival and propagation is very important. The release of pro-inflammatory cytokines and chemokines and the infiltration of immune cells constitute the innate response against influenza viruses, which eventually leads to adaptive humoral and cellular influenza-specific responses (Schmolke and Garcia-Sastre, 2010; Asha et al., 2021). The understanding of how influenza viruses are able to evade these responses has been made easier through the use of reverse genetics. The knowledge accruing from these studies in turn become very useful in the design of modified influenza viruses that are able to overcome the viruses defense mechanisms and generate immunity that is effective in neutralizing the pathogenic virus. The goal of researchers is to design a universal influenza vaccine that is able to produce cross-neutralizing immune response targeting the highly conserved internal proteins of the virus and able to induce effective cytotoxic immunity against any influenza strain. This is achievable through the understanding of epitope immuno-dominance, a study that is also aided by the use of reverse genetics.

### 6.3 Understanding the drug-resistance mechanisms of influenza viruses

Resistance to some antiviral drugs by influenza viruses has been reported, for example, against the NA inhibitor zanamivir and the M2 blocker amantadine (Whitley et al., 2013; Yen et al., 2013). There is the need to understand the mechanisms of the reported resistance in order to design new drugs that can factor in these resistance factors and be more effective. This understanding is made easier through the use of reverse genetics tools.

### 6.4 Viral vaccine vectors and immunotherapies

Vectors are becoming increasingly important in vaccine development and immunotherapies. The current deeper understanding of influenza virus has made it possible to use the modified strains for this purposes, and especially in cancer immunotherapy (Restifo et al., 1998). The same tools are being applied in vaccines against HIV/AIDS (Li et al., 1993a), malaria (Li et al., 1993b) and certain bacterial diseases (Gilleland et al., 1997). All this is made possible by the advances in the use of reverse genetic applications. Since the organisms are constantly evolving, however, there is the need to continue advancing the tools available through reverse genetics and expanding the body of knowledge for the benefit of mankind and animals.

## 7 *In vitro* studies of influenza anti-infective

The influenza virus polymerase acidic (PA) protein, essential in viral RNA transcription and viral replication, is a promising antiviral target. RO-7 is a novel PA protein endonuclease inhibitor (Jones et al., 2017) that has been studied for its potential role in inhibiting influenza A and B viruses in Madin-Darby Canine Kidney (MDCK) and multiple influenza strains A (H1N1, H3N2, H5N1, H7N9, H9N2) and B viruses. A lethal BALB/c mouse model of RO-7 using 5MLD_50_ of A/California/04/2009 A(H1N1)pdm09 or B/Brisbane/60/2008 showed increased survival, reduced viral load in the lungs, decreased morbidity, and reduced lung pathology compared to controls (McKimm-Breschkin et al., 2018).

A Japanese research team demonstrated that the transmembrane Ca^2+^ channel is the key receptor for influenza A virus (IAV). In laboratory studies using cultured human cells, IAV binds to Ca^2+^ channels to trigger an influx of Ca^2+^, viral entry and infection while inhibition of the Ca^2+^ channels prevent IAV-induced Ca^2+^ influx and entry of the virus. Using mice models, the team found a dose-dependent reduction in the number of replicated viral progeny when administered with calcium channel blockers (CCB) intranasally. When infected with higher amounts of IAV, CCB prolonged survival while the untreated group died within 5 days of infection (Fujioka et al., 2018).

Polyclonal antibodies, derived from either hyperimmunized animals or humans, have been utilized to prevent or treat several infectious diseases. The transchromosomic (Tc) bovine platform technology is able to rapidly create fully human immunoglobulins using a triple knockout of bovine immunoglobulin (Ig) genes and replacement with human Ig genes (Dye et al., 2016). In a murine model of influenza A(H1N1)pdm09 virus, the dosage of SAB-100 (Tc-bovines hyperimmunized with a tri-valent seasonal influenza split virion) was evaluated. A single prophylactic dose of SAB-100 at 1.33, 4, or 12 mg/kg provided 100% protection. A single therapeutic dose with 12, 24, or 48 mg/kg dose of SAB-100 provided 100% protection. A study using another animal model investigated the effect of sub-therapeutic doses of SAB-100 (1.5 mg/kg) and oseltamivir (0.3 mg/kg) either alone or co-administered at 12 hours after infection. Mice administered the with combination therapy had 90% survival, while 60% survival was observed in mice that received SAB-100 alone, and 40% survival was observed in mice that received oseltamivir alone (McKimm-Breschkin et al., 2018).

## 8 Clinical trials and outcomes of influenza therapeutics

### 8.1 Intravenous zanamivir

From 2011 - 2015, clinical trials to evaluate the safety and efficacy of intravenous zanamivir (IVZ) in treating hospitalized patients with severe influenza were done. For the Phase II clinical trial, patients with confirmed influenza were given 600 mg IVZ twice a day (adults) or weight-based or age-adjusted dose twice a day (pediatrics) for 5 to 10 days (Marty et al., 2014). For the Phase III studies, patients with confirmed influenza enrolled within 6 days of flu illness were given 300 IVZ or 600 IVZ twice a day for 18 days or oseltamivir (OS) 75 mg twice a day for 5 - 10 days. The study showed an approximate 0.5-day improvement in time to clinical response (vital sign resolution, hospital discharge) in the 600 mg IVZ group compared to oseltamivir (not significant, not superior), but there was no difference in the median change in viral load (Marty et al., 2017). In a study previously reported at Options for the Control of Influenza- IX, 133 pediatric patients in Japan aged between 4 and 12 years old that presented within 48 hours of flu illness were randomly given one of four: oral oseltamivir, inhaled zanamivir, intravenous peramivir, or inhaled laninamivir. Peramivir had shorter time for viral clearance when compared to oseltamivir (adjusted P = 0.035) but there were no significant differences in HAI titer increase between treatments (McKimm-Breschkin et al., 2018).

### 8.2 Umifenovir

Umifenovir (Arbidol), a fusion inhibitor specifically targeting hemagglutinin, is licensed in Russia and China for treatment and prophylaxis of both influenza A and B. Umifenovir produces antigen-specific antibodies and is able to induce interferon-production and stimulate macrophages (Glushkov et al., 1999). Retrospective observational studies done in 2010–2011 (Leneva et al., 2009) and 2014–2015 flu seasons with 5,287 hospitalized influenza patients were given 4 × 200 mg umifenovir or 2 × 75 mg oseltamivir within 48 hours of onset of flu symptoms. Fever duration in patients treated with umifenovir (< 2 years old) was 4.13 days and 2.45 days (> 65 years old) as compared to 3.67 and 4.27 for age-matched controls. No significant differences were seen in the duration of illness and main symptoms of influenza between the umifenovir and oseltamivir treated groups.

### 8.3 Pimodivir

Pimodivir is a first of its kind inhibitor of the PB2 subunit of IAV polymerase complex developed to treat IAV including H1N1 (pandemic) and H5N1 (avian) strains (Fu et al., 2016). A Phase II study evaluated the safety and efficacy of pimodivir in adults 18 - 65 years of age with uncomplicated IAV infection who presented with less than 48 hours of symptom onset. Patients were randomly given placebo, pimodivir 300 mg, pimodivir 600 mg, or the combination pimodivir 600 mg/oseltamivir 75 mg for 5 days. Pimodivir 600 mg resulted in a greater decrease in qRT-PCR area under the curve (AUC) for viral load from day 1–8, compared to 300 mg [-4.5 (−8.0; −1.0); 0.012 vs −3.6 (−7.1; −0.1); 0.044 log_10_ copies/ml]. The pimodivir and oseltamivir treatment showed further reduction when compared to pimodivir 600 mg (−4.1 log_10_ copies/mL, −7.4 to −0.7, P = 0.017) but the difference was not associated with improved clinical benefit. The dosage 600 mg was selected for Phase III studies (McKimm-Breschkin et al., 2018). In March 2017, the US Food and Drug Administration granted pimodivir Fast Track Designation due to its potential for treatment of influenza A infections (Finberg et al., 2019).

### 8.4 Oseltamivir, amantadine, and ribavirin

Triple combination of oseltamivir, amantadine, and ribavirin was synergistic and effective against several influenza A virus subtypes (H1N1, H3N2, and H5N1) (Nguyen et al., 2009). A randomized, blinded, multi-center Phase II study designed to enroll participants that were either 65 years of age or older, had a chronic medical condition, and/or were obese with confirmed influenza A or B, were randomly administered either oseltamivir alone OR the combination of oseltamivir, amantadine, and ribavirin for 5 days. The primary endpoint of the study was the percentage of participants with virus detectable by PCR in a nasopharyngeal swab at day 3. Eighty of 200 (40.0%) participants in the combination arm had virus detectable at day 3 compared to 97 of 194 (50.0%) (95% C.I. 0.2–19.8%, P = 0.046) in the control arm, but the difference was not associated with clinical benefit (McKimm-Breschkin et al., 2018).

### 8.5 Danirixin

Danirixin (DNX), also known as GSK1325756, is an oral selective, competitive reversible inhibitor of CXC chemokine receptor 2 (CXCR2) developed as an anti-inflammatory agent for the treatment of severe asthma and chronic obstructive pulmonary disease (COPD) (Busch-Petersen et al., 2017). Because CXCR2 is a key receptor in the chemotaxis of neutrophils to areas of inflammation, DNX is also in development for uncomplicated influenza and for intravenous (IV) therapy for patients hospitalized with influenza. A randomized double-blind placebo-controlled clinical trial in outpatients with uncomplicated PCR-confirmed influenza evaluated the safety and tolerability of 75 mg DNX with and without 75 mg of oseltamivir given twice daily for five days. In all treatment groups, mean peripheral neutrophils decreased from baseline to day 3 and resolved by day 8 with a trend toward shorter median times to resolution of all influenza symptoms in the DNX + oseltamivir group (112 hours) than in the oseltamivir only group (267 hours). A study on hospitalized flu patients is underway (NCT02927431).

### 8.6 Baloxavir marboxil

Baloxavir marboxil (S033188) is a potent, selective small molecule inhibitor of the cap-dependent endonuclease that is essential during viral mRNA biosynthesis of influenza A and B viruses. The active form, S-033447 (Uehara et al., 2016), can inhibit viral mRNA synthesis thereby preventing protein production of the flu virus. In the Phase II trial, influenza-confirmed adult patients were given a single dose of 10, 20 or 40 mg or placebo and a quick decrease in the viral load of 3 to 4 log_10_ by 24 hours was observed with earlier resolution of fever and all symptoms compared to the placebo group (mean ∼50 vs. 77 hours). In addition, a negative correlation was observed between drug dose and a change in virus titers at day 2 and day 3 (McKimm-Breschkin et al., 2018). The drug was approved in 2018 by the US FDA and Japan’s Ministry of Health, Labour and Welfare for treatment of uncomplicated influenza in patients aged 12 years and older. In a randomized placebo-controlled phase III trial on over 2,000 patients, baloxavir showed a superior efficacy in a single dose to placebo and similar efficacy to oseltamivir for reducing influenza symptoms in the high-risk outpatients (Ison et al., 2020).

### 8.7 Nitazoxanide

In two completed randomized double-blind placebo-controlled Phase III trials (NCT01610245), oral NTZ 600 mg given twice daily alone for 5 days was compared with NTZ in combination with oseltamivir, and with oseltamivir alone. Study showed no difference in the time to resolution of symptoms between the NTZ, oseltamivir, and NTZ and oseltamivir combination vs. placebo (McKimm-Breschkin et al., 2018).

### 8.8 Monoclonal antibodies

A growing pipeline of broadly neutralizing antibodies is being developed in people exposed to infectious diseases, including influenza, COVID-19 and respiratory syncytial virus (RSV). Several combinations may potentially be able to protect against pandemic strains of respiratory viruses. MHAA4549A, a human immunoglobulin G1 (IgG1) monoclonal antibody, can bind to a highly conserved epitope on the stalk of IAV hemagglutinin (HA) and block the HA-mediated membrane fusion in the endosome, promoting antibody-dependent cellular cytotoxicity (ADCC) of the infected cell. It can neutralize all known human IAV strains (Gupta et al., 2016). In a Phase II study, subjects were given single-dose of either 400, 1200, or 3600 mg of MHAA4549A intravenously up to 36 hours after inoculation with IAV (McBride et al., 2017; Deng et al., 2018). No PK drug–drug interaction between MHAA4549A and oseltamivir was observed (Deng et al., 2018) and only the 3600 mg group showed significantly reduced viral burden by RT-PCR and TCID_50_ assay (McBride et al., 2017).

## 9 Conclusion

Antivirals and chemotherapeutics are essential complement in clinical management of influenza. Some drugs discovered many years ago may still be useful but of interest is the emergence of novel drugs and drug combinations that has proven very effective. Outcome of clinical management of influenza showed that early treatment with antivirals can significantly bring down the risk for severe illness or death. Thus, influenza epidemic and pandemic can be mitigated with a combination of viral chemotherapeutics in addition to prior vaccination and other public health measures.

## Author contributions

CM: Conceptualization, Writing – original draft, Writing – review & editing. MS: Writing – original draft, Writing – review & editing. KA: Writing – original draft, Writing – review & editing. LS: Writing – original draft, Writing – review & editing. BK: Conceptualization, Resources, Visualization, Writing – original draft, Writing – review & editing.
